# Medical Men, Medical Papers, and Insurance

**Published:** 1907-11-16

**Authors:** 


### The Hospital

A Journal of

The Medical Sciences and Hospital Administration.

New Series. No. 34, Vol. II. [No. 1106, Vol. XLIII.] SATURDAY, NOVEMBER 16, 1907.

### Medical Men, Medical Papers, and Insurance.

We have always held the opinion that the ideal medical newspaper should be conducted upon lines which enable the readers and the proprietors to co-operate in its development, and to share in its success. In accordance with this principle, and recognising that Combination can effect so much in the way of advantage in the present day, we have felt impelled to use our influence and knowledge to bring about a system of co-operation between one of the strongest and best of English assurance companies and those members of the medical profession who choose to avail themselves of it. This system enables practitioners who are regular subscribers to The Hospital to obtain a policy covering accidents and certain diseases, to which they may be especially liable in the ordinary course of their day's work, at a premium of one pound a year, only. The plan is fully explained in a letter and prospectus from the Secretary of The Employers' Liability Assurance Corporation, Limited, which we publish this week as a special Supplement. The benefits are confined to registered members of the medical profession who reside in the British Islands, and the plan has the further advantage that the Assurance Company agrees to consider the inclusion of further risks not yet covered by "The Doctor's Own Policy." Those members of the profession who take out this policy are invited to test the scheme as it stands, and to indicate during the year any further risks which, if covered, would tend to make the policy an ideal one for medical practitioners. We take it that every practitioner will be glad to possess a policy of the kind, with the prospect of eventually covering every risk which specially attaches to those who habitually follow the practice of medicine. It remains to be seen if this scheme of co-operation between this great insurance company, the members of the medical profession, and the proprietors of The Hospital, will be welcomed, as we believe it will be, by the large body of medical men.

